# Aberrant imprinting may underlie evolution of parthenogenesis

**DOI:** 10.1038/s41598-018-27863-7

**Published:** 2018-07-13

**Authors:** Olga Kirioukhova, Jubin N. Shah, Danaé S. Larsen, Muhammad Tayyab, Nora E. Mueller, Geetha Govind, Célia Baroux, Michael Federer, Jacqueline Gheyselinck, Philippa J. Barrell, Hong Ma, Stefanie Sprunck, Bruno Huettel, Helen Wallace, Ueli Grossniklaus, Amal J. Johnston

**Affiliations:** 1 University of Heidelberg, Centre for Organismal Studies, Laboratory of Germline Genetics & Evo-Devo, Heidelberg, Germany; 2 Jacobs University, Life Sciences & Chemistry, Laboratory of Germline Genetics & Evo-Devo, Bremen, Germany; 3 University of Zurich, Department of Plant and Microbial Biology and Zurich-Basel Plant Science Center, Zurich, Switzerland; 4 The Pennsylvania State University, the Huck Institute of Life Sciences, Department of Biology, The University Park, Pennsylvania, USA; 5 Fudan University, State Key Laboratory of Genetic Engineering, Institute of Plant Biology, School of Life Sciences, Fudan University, Shanghai, China; 6 University of Regensburg, Cell Biology and Plant Biochemistry, Regensburg, Germany; 7 Max-Planck-Institute for Plant Breeding, Cologne, Germany; 8 University of the Sunshine Coast, Faculty of Science, Health, Education and Engineering, Genecology Research Centre, Maroochydore, Australia; 9 ETH Zurich, Department of Biology and Zurich-Basel Plant Science Center, Zurich, Switzerland; 10 Present Address: University of Agricultural Sciences, College of Agriculture Sciences, Department of crop physiology, Hassan, India; 11 Present Address: New Zealand Institute for Plant and Food Research, Christchurch, New Zealand

## Abstract

Genomic imprinting confers parent-of-origin-specific gene expression, thus non-equivalent and complementary function of parental genomes. As a consequence, genomic imprinting poses an epigenetic barrier to parthenogenesis in sexual organisms. We report aberrant imprinting in *Boechera*, a genus in which apomicts evolved from sexuals multiple times. Maternal activation of a MADS-box gene, a homolog of which is imprinted and paternally expressed in the sexual relative *Arabidopsis*, is accompanied by locus-specific DNA methylation changes in apomicts where parental imprinting seems to be relaxed.

## Introduction

Genomic imprinting refers to epigenetic gene regulation that leads to the parent-of-origin-specific expression of alleles, and it was proposed to differentially control offspring development^reviewed in^ ^1^. Because genomic imprinting causes parental genomes to be non-equivalent, it prevents parthenogenetic embryo development by enforcing contribution of both parental genomes^2^. In flowering plants, most imprinted genes are mono-allelically expressed in the embryo-nourishing endosperm tissue, and a few are imprinted in the embryo^reviewed in ^^3^. Imprinting mechanisms may also serve as barriers to inter-specific or inter-ploidy hybridization in sexual plants, perhaps as a sensor that detects the correct parental gene dosage^reviewed in^ ^4^. In contrast, apomicts can tolerate a skewed parental genome constitution, e.g. absence of the paternal genome in the embryo and sometimes an altered parental genome dosage in the endosperm^5,6^.

DNA and histone methylation play predominant roles in genomic imprinting. An imprinted and paternally expressed MADS-box gene encoding the transcription factor PHERES1 (PHE1) promotes embryo growth and is maternally repressed by a H3K27me3 histone methyltransferase MEDEA (MEA), which restricts growth in the sexual species *Arabidopsis thaliana*^4,7^. The contrasting imprinting effects between *MEA* and *PHE1* lend support to the parental offspring theory^6^. Since parthenogenetic embryos lack a direct paternal contribution, we hypothesized that a relief of imprinting may have played a role in the evolution of parthenogenesis in plant species that reproduce asexually through seeds via apomixis. The *Boechera* genus is closely related to *Arabidopsis*, belonging to the same major clade within the Brassicaceae phylogeny^8^, and it consists of both sexual and apomictic (parthenogenetic) populations^9^. The genetic basis of parthenogenesis in *Boechera* is currently unknown. Here, we asked whether changes in the status of imprinting are involved in parthenogenesis in *Boechera*. For this, we analysed the spatio-temporal expression pattern and DNA methylation status of the *Boechera* homolog of *PHE1*, which is a paternally-expressed imprinted gene in *Arabidopsis*. We examined a diploid sexual *B*. *stricta* (*Sex-1*) and a triploid apomict *B*. *gunnisoniana* (*Apo-1*) using cytological and molecular approaches. A closely related diploid apomict, *B*. *divaricarpa* (*Apo-2*)^10^, was included for additional comparisons, in order to eliminate ploidy effects.

**Figure 1:**
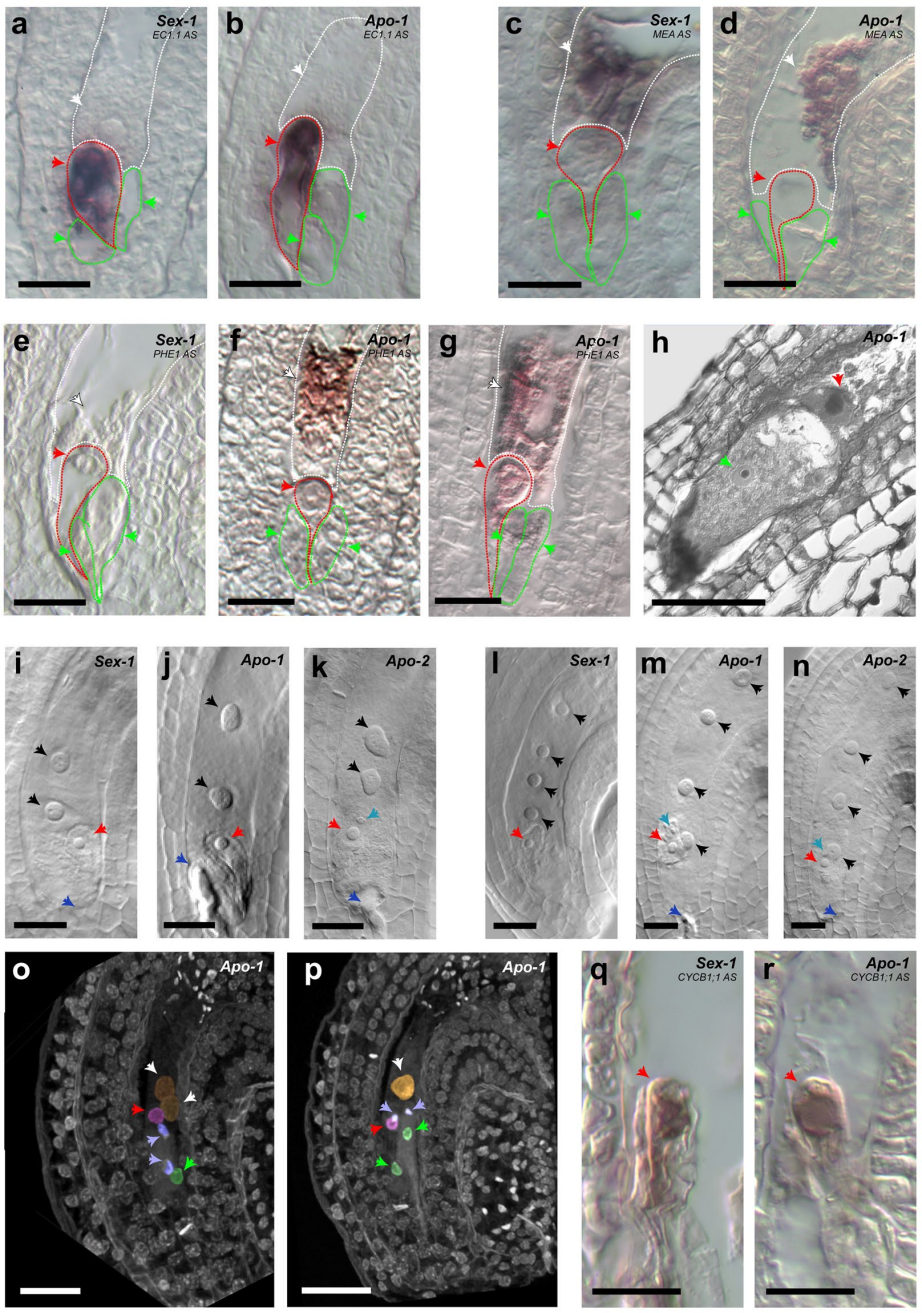
Pseudogamous parthenogenesis in *Boechera* is accompanied by sexual-like gene expression patterns and deregulation of a MADS-box gene. (**a**,**b**) Heterologous mRNA *in situ* signals of *EC1*.*1* in sexual *versus* parthenogenetic egg cells. Arrow-heads: red – egg cell, green – synergids, white – central cell nuclei. (**c**,**d**) *MEA* signals in central cells. (**e**–**g**) Heterologous signals of *PHEL1* in apomictic *versus* sexual embryo sacs. (**h**) An apomictic egg cell of *Apo-1* (red arrow-head) at 3 days after emasculation. (**i**–**n**) Fertilized ovules (dark-blue arrow-heads – pollen tube entry, black arrow-heads – endosperm). (**m**,**n**) An unfused sperm nucleus (light-blue arrow-head) is visible proximal to the parthenogenetic egg cell (red arrow-heads). (**o**,**p**) Confocal micrographs of *Apo-1* ovaries at fertilization. (**o**) Two sperm cells (light-blue arrow-heads) discharged into an apomictic embryo sac. (**p**) Sperm cell arrival coincides with polar nuclei fusion (white arrow-head). (**q**,**r**) *CYCB1;1* mRNA *in situ* signals at one-celled embryo stage (red arrow-heads). Scale bars in (**a-r**) 20 µm.

Apomictic reproduction requires two major alterations of the sexual pathway: meiosis is avoided to generate unreduced gametes, followed by parthenogenesis enabling embryo development without a paternal contribution^5^. In apomictic *Boechera*, both female and male meioses are equally circumvented (Supplementary Figs 1 and 2 and explanations therein) and the female and male gametogenesis produce unreduced egg and sperm cells, respectively (Supplementary Fig. 3). In *Arabidopsis*, EGG-CELL 1.1 (EC1.1) peptides accumulate in the egg cell prior to fertilization and prevent multiple sperm fusions likely through male-female signalling processes^11^. Abundant transcripts of *EC1*.*1* were detected in *Boechera* by heterologous mRNA *in situ* hybridization with an *Arabidopsis* probe. The *Boechera EC1.1* was expressed in the egg cell before fertilization in the sexual *Sex-1* line as well as at the onset of parthenogenesis in the apomictic *Apo1* line (Fig. 1a,b). This likely reflects the requirement of egg-sperm signalling in the apomict similarly to the sexual *Arabidopsis*^11^. Preventing pollination in *Apo-1* did not lead to parthenogenetic embryo development (Fig. 1h), further supporting the view that some aspects of fertilization are necessary for parthenogenesis in *Boechera*. In the sexual *Sex-1* as well as the apomict *Apo-1*, the central cell exhibited weak but detectable signal of *MEA* transcripts (Fig. 1c,d) similar to *Arabidopsis*^12^, in which it has been shown to prevent autonomous divisions in the central cell^13^. In both the sexual *Sex-1* and the apomicts, *Apo-1* and *Apo-2*, the pollen tube enters the embryo sac, and the two sperm cells each target the egg and central cell, respectively (Fig. 1i–p). Regardless of the reproductive mode, nuclear fusion occurs between one of the two sperm cells and the central cell, which is followed by primary endosperm divisions (Fig. 1i–n). However, unlike egg-sperm karyogamy leading to zygote formation in the sexual (Fig. 1i,l), the second sperm nucleus in the apomicts persisted in the vicinity of the nucleus of the egg but no fusion occurred even at a stage when mitotic divisions in the endosperm advanced (Fig. 1k,m–p). This observation suggests that presence of the sperm near the egg cell of the apomict might serve as activation source to induce pseudogamous parthenogenesis. The resulting parthenogenetic embryos were indistinguishable from the sexual ones in terms of morphology and expression of the cell-division marker gene *CYCB1;1* (Fig. 1q,r). Furthermore, self-pollination in *Boechera* apomicts seems to be required for maintaining genome integrity in the progeny. The majority of the individuals arising from inter-specific pollination between apomicts displayed a range of genomic alterations and partial breakdown of genome integrity (Supplementary Fig. 4 and discussions therein). Some of the progeny that was of solely maternal genotype (i.e. of parthenogenetic origin) upon inter-specific pollination exhibited an array of vegetative and reproductive defects including self-incompatibility, which was not observed in the apomictic self-progeny. Global epigenetic changes in the maternal genome could possibly account for such morphological aberrations in clonal offspring. Collectively, our observations suggest that male cues from the self-parent are likely essential at fertilization for the initiation of pseudogamous parthenogenesis, and might be necessary for the maintenance of epigenetic states.

**Figure 2:**
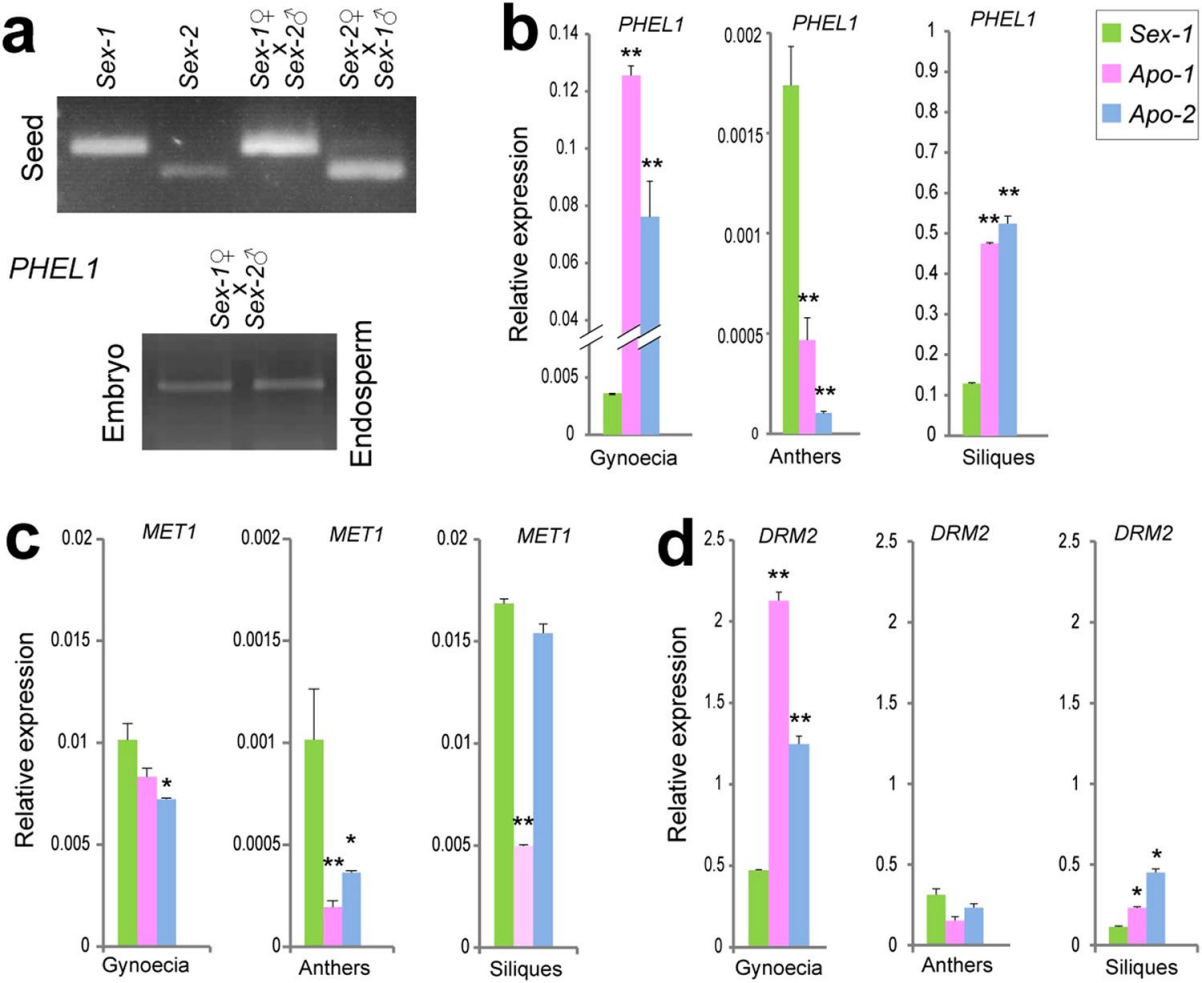
The imprinted *PHEL1* is upregulated in maternal, female gametophytic, and sporophytic tissues of apomictic *Boechera*, correlating with deregulation of DNA methyltransferase genes. (**a**) Maternal and paternal *PHEL1* transcripts assayed by allele-specific RT-PCR in immature seeds, embryo, and endosperm fractions upon reciprocal crosses between two sexual *Boechera* accessions. (**b**–**d**) Relative transcript levels of *PHEL1*, *MET1*, and *DRM2*. Expression deviation from sexual *Boechera* (t-test significance levels: **α ≤ 0.01;*α ≤ 0.05).

To understand the impact of apomictic mode of development on genomic imprinting we analysed the *PHERES1* locus *of Boechera*. In *Arabidopsis*, the maternal allele is silenced by MEA, but the paternal allele is expressed during seed development^14,15^. Following incompatible hybridizations, the maternal *PHE1* allele can be de-repressed^4,16^. We identified two *Boechera* homologs of *PHE1*: *PHERES-LIKE 1 (PHEL1)* and *PHEL2*. PHEL1 has up to 64% amino acid sequence identity with *Arabidopsis* PHE1 or PHE2 (Supplementary Fig. 5). *PHEL2* represents a pseudo-gene without detectable expression (Supplementary Fig. 6a). In order to characterize the imprinting status of *PHEL1* in *Boechera* sexuals and distinguish the maternal and paternal alleles, we used another sexual diploid species, *B*. *perennans* (*Sex-2*) offering a sequence polymorphism in *PHEL1*. In reciprocal crosses between Sex-1 and Sex-2, we analyzed allele-specific expression of *PHEL1* by RT-PCR experiments. Only the maternal allele of *PHEL1* is expressed in the embryo and endosperm tissues of the *Boechera* sexuals (Fig. 2a, Supplementary Fig. 6b,c), unlike its *Arabidopsis* counterpart *PHE1*^14^. It is likely that expression of the maternal *PHEL1* allele results from a switch in the state of imprinting within sexual *Boechera* species, which likely arose in response to hybridization-driven speciation and subsequent genome modifications as previously proposed^17^.

In sexual *Boechera* species, *PHEL1* is expressed at very low levels both in the female (gynoecia) and male (anthers) reproductive organs, yet expression in the mature female tissues is nearly three-fold greater than in the male (Fig. 2b). *PHEL1* expression could not be detected *in situ* in sexual ovules due to its low abundance, but the levels of the corresponding transcripts were quantified by qRT-PCR. *PHEL1* transcripts were abundant in the gynoecia and siliques of *Apo-1* and *Apo-2*; the asexual gynoecia had up to 40-fold higher *PHEL1* expression levels compared to the sexual ones irrespective of the ploidy level of the apomict (Fig. 2b). In addition, faint but specific *in situ* expression of *PHEL1* was detected in the apomictic embryo sac of *Apo-1* (Fig. 1f,g, compare to *Sex-1* in Fig. 1e and to the sense probe in Supplementary Fig. 6d). *PHEL1* expression in the apomicts showed 250–400-fold higher transcript levels in female than male floral organs and in the embryos compared to the levels of expression in the sexual (Fig. 2b). We propose that these high levels of the maternal *PHEL1* transcript prior and during embryo development may play a role in parthenogenesis.

In *Arabidopsis*, METHYLTRANSFERASE 1 (MET1) is pivotal for maintenance of CG DNA methylation, while CHROMOMETHYLASE 3 (CMT3) maintains non-CG-methylation; and DOMAINS-REARRANGED METHYLTRANSFERASEs 1 and 2 (DRM1/2) control RNA-dependent DNA methylation (RdDM) *de-novo* in all contexts^18^. Their function is thought to be critical for epigenetic reprogramming and genomic imprinting during gametogenesis and seed development^3^. When we examined genes expression levels of the corresponding *Boechera* homologs, we noticed a complex situation with respect to common and/or taxon-specific expression patterns of genes coding for DNA methyltransferases (Fig. 2c,d, Supplementary Fig. 7). In apomicts, *MET1* was marginally lower in gynoecia, and significantly down-regulated in anthers, in comparison to the sexual lines (Fig. 2c). This situation persisted after fertilization in *Apo-1*. *DRM2* was significantly upregulated in gynoecia and siliques of both apomicts (Fig. 2d). In *Arabidopsis*, DNA methylation at the *PHE1* locus is regulated by cytosine methylation machinery involving MET1 and DRM2, and influences its parental expression levels^15^. Taken together, reduced *MET1* and increased *DRM2* levels in female floral tissues might provide an explanation for high levels of maternal *PHEL1* in *Boechera* lines. Furthermore, in seedlings of the *Boechera* apomicts, increased *PHEL1* expression also correlated with upregulation of *DRM2* and down-regulation of *MET1* (Supplementary Fig. 7d–f). Although cell-type-specific comparative gene expression and DNA methylation profiling remains to be elucidated (and poses a significant challenge in non-model systems like *Boechera*) we propose that MET1/DRM2-mediated DNA methylation changes might be responsible for the elevated expression of *PHEL1* in *Boechera* apomicts.

**Figure 3:**
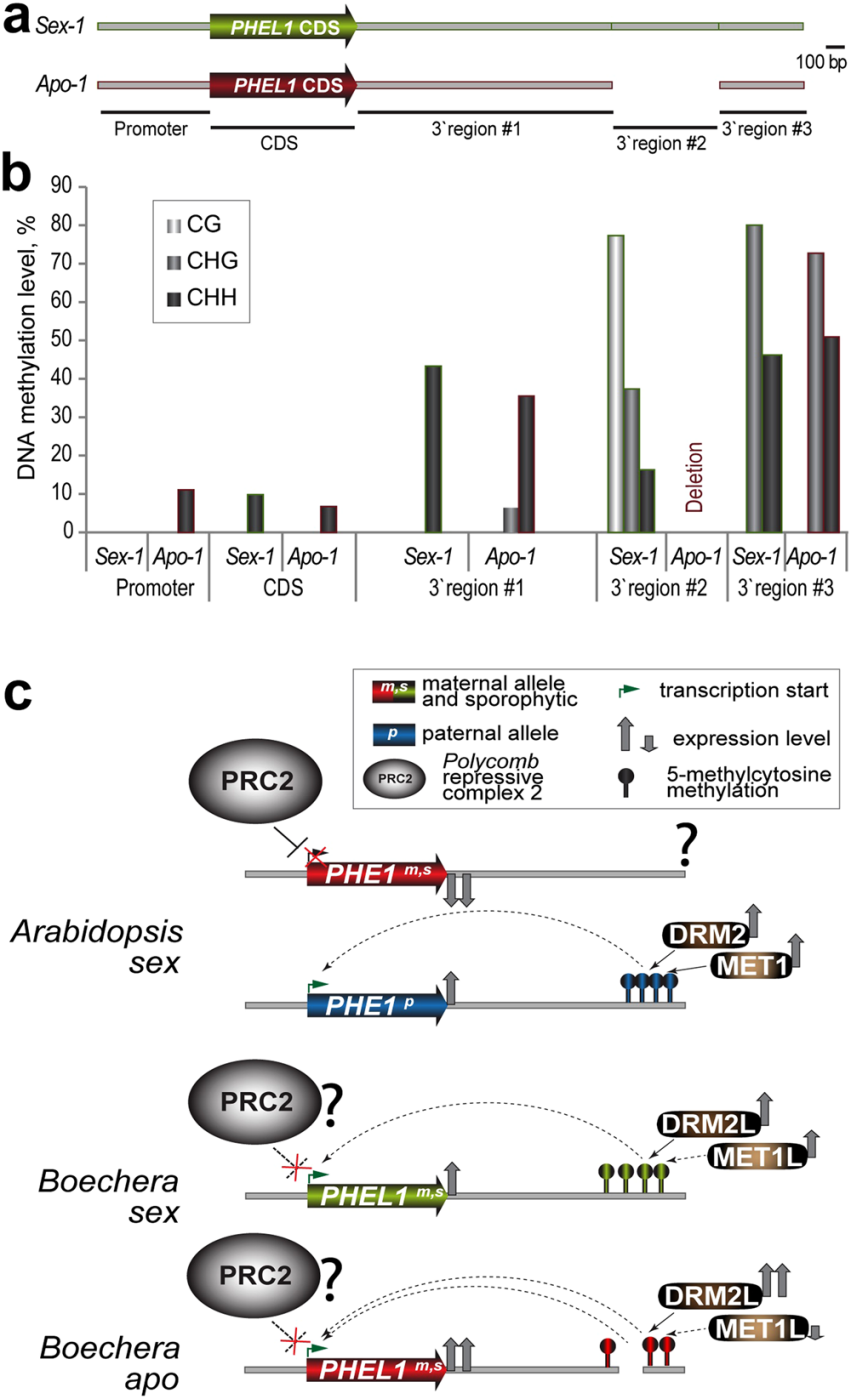
DNA methylation analysis of the *PHEL1* loci in a sexual and an apomictic *Boechera* line, and a proposed epigenetic model of *PHEL1* regulation in sexual (*sex*) *versus* apomictic (*apo*) lines. (**a**,**b**) Scheme of *PHEL1* loci and corresponding percent methylation identified by bisulfite-sequencing. (**c**) An illustration of the proposed epigenetic regulation of *Arabidopsis PHE1* versus *Boechera PHEL1* by DNA/histone methylation. PRC2, *Polycomb* Repressive Complex 2 containing the MEA histone methyltransferase.

We tested by bisulfite sequencing whether the active maternal *PHEL1* allele in apomictic *Boechera* exhibits DNA methylation footprints distinct from those in the sexual. We found that the most distal 3′ region ca. 2 kb downstream of the *PHEL1* gene showed strong DNA methylation in a non-CG context in both sexual and apomictic gynoecia (3′-#3, Fig. 3b). Intriguingly, we found that a 0.6 kb DNA fragment distal to the *PHEL1* gene (3′-#2) is present only in the *Sex-1* line, and is heavily methylated primarily in CG but also in non-CG contexts (Fig. 3a,b). This methylated region (3′-#2, or 3′MR) consisted of several repeats (Supplementary Fig. 8). The 3′MR was absent in an *Apo-1 PHEL1* allele; a similar deletion was also found in *Apo-2*. In brief, a heavily methylated distal DNA fragment was absent in two apomict-specific *PHEL1* alleles but present in a sexual, and this deletion positively correlated with elevated *PHEL1* expression in the apomicts.

Apomixis is reported in only about 0.5% of the Brassicaceae genera, which mostly occur in extreme environmental conditions (discussed in^10^). In particular, the North-American *Boechera* species are likely to have arisen from millions of years of evolutionary bottle-necks and reticulate evolution. Hybridization between sexual *Boechera* genotypes may have paved the way for genomic imprinting to become relieved from tight control, based on a genomic landscape with two contrasting genomes^17,19^. Ultimately, some *Boechera* hybrids may have had an epigenetic environment conducive for the evolution of novel apomictic traits, such as parthenogenesis. Existence of multiple independently evolved apomictic *Boechera* population allow us to propose that some convergent (epi)genetic mechanisms may play a prominent role here. Our findings suggest that parent-of-origin expression of *PHEL1* or *PHE1*^15^ across genera is highly correlated with DNA methylation pattern of the corresponding loci*;* however, this regulation is modified in *Boechera* in terms of a) reversion of the imprinting status resulting in expression of the maternal *PHEL1* allele; and b) deletion of the heavily methylated 3′MR in the alleles specific to apomicts, with a concomitant increase in expression. Figure 3c proposes a model where distinct epigenetic regulation of *PHE1*/*PHEL1* based on DNA methylation may have enabled parthenogenesis to evolve in *Boechera*. Our findings in *Boechera* show similarity to an artificially induced case of parthenogenesis in mice^20^, where loss of distal DNA methylation causing maternal activation of the paternally expressed *Insulin-like growth factor 2* (*Igf2*) gene was sufficient to induce parthenogenesis. We thus propose that alterations in the control of genomic imprinting enable the adjustment of parental gene dosage necessary for parthenogenesis to evolve.

## Materials and Methods

### Plant material and growth conditions

Diploid sexual and/or triploid asexual *Boechera* seeds were kind donations from various sources^10^. Both triploid *Apo-1* and diploid *Apo-2* were first analyzed for ploidy by bulked seed flow-cytometry^10^, which revealed the presence of occasional 6 C (hexaploid) and 4 C (tetraploid) embryo peaks, respectively. The offspring seedlings were ploidy-analyzed using a FacsCantoII cytometer (BD Biosciences, USA), and rare hexaploid *Apo-1* plants were eliminated from further analyses. Single seed flow-cytometry gave an over-estimate of apomeiosis per plant as only the fertile seeds were taken for analyses; therefore, expressivity of apomeiosis and parthenogenesis was determined by individual seed ploidy analyses by flow-cytometry, and subsequently by ovule clearing and seed counts^10^. Plants were grown under long-day conditions at 18–21^o^C.

### RNA extraction, cDNA synthesis, real-time qRT-PCR

RNA isolation and cDNA synthesis were performed as described in^10^. Locus-specific fragments across all *Boechera* strains were PCR-amplified based on conserved *Arabidopsis* and *Boechera* sequences available from public repositories (NCBI, http://www.ncbi.nlm.nih.gov; Phytozome, http://phytozome.jgi.doe.gov) and sequenced. We cloned the entire *PHEL1* and *PHEL2* loci in all *Boechera* species analysed here based on a *Sex-1*-specific template. Allele-specific *PHEL1* transcript fragments were genotyped upon *Bst*UI digestion (New England Biolabs, USA). qRT-PCR SYBR Green assays were performed in a StepOnePlus Real-Time-PCR System (Applied Biosystems, USA) with three biological replicates and normalized as described^10^ using *RPS18* gene as a reference. Primer sequences are given in Supplementary Table 1.

### DNA methylation analysis by bisulfite sequencing

DNA methylation conversion was performed using EpiTect Bisulfite kit (Qiagen, Germany). For each library, 8–10 egg cell-containing gynoecia at the stage just prior fertilization were processed according to the manufacturer instructions yielding two BS-seq libraries (*Sex-1* 120 ng and *Apo-1* 200 ng). Library was sequenced using standard Illumina 2500 pipeline at the MPIZ Genome Centre, Cologne, Germany. In brief, library quality check was performed with FastQC method. BS-seq was set to 6 Gb for *Sex-1* and 18 Gb for *Apo-1* aiming 30 × genome coverage. Conversion efficiency was evaluated using Bismark alignment and methylation caller^21^. Detected genome-wide methylation levels of cytosines in the CG, and non-CG contexts were 19.6%,and 8.1%, respectively, which was similar to *Arabidopsis*^22^ and indicated a very good level of bisulfite conversion; the high conversion efficiency was further confirmed by detecting long stretches (ca. 0.5–1 kb) of fully bisulfite converted DNA. Sequence reads were mapped to the corresponding *PHEL1* sequences from *Sex-1* and *Apo-1* using Bismark platform.

### Heterologous *in situ* mRNA hybridization

*Arabidopsis*-specific heterologous *in situ* probes were prepared from corresponding cDNA clones; and mRNA *in situ* hybridization^23^ was modified to include an additional RNase A treatment step (20 µg/ml for 30 min incubation at 37 °C) to remove unspecific background signals. The mRNA *in situ* mRNA hybridization worked efficiently, particularly when the transcripts were abundant. For *MEA* and *PHE1* RNA probes, it was necessary to add hybridization solution on slides during each day for up to three days to enhance its very weak signal; the specificity of probe binding was ensures by RNase A treatment (see above). In the case of probing against *PHERES*-like genes in *Boechera*, although the *in situ* probe used cannot distinguish gene-specific transcripts due to a high degree (~80%) of nucleotide identity between *PHEL1* and *PHEL2*, we are confident that we indeed detected *PHEL1*-specific transcripts *in situ* because *PHEL2* signals were barely detectable even in qRT-PCR assays. Ovule and seed clearing, DIC and confocal microscopy upon propidium-iodide staining, and image analyses using Imaris (Bitplane, Switzerland) were performed as described^24,25^.

## Electronic supplementary material

Supplement
