# Supplementary material for: Aberrant imprinting may underlie evolution of parthenogenesis
Source: Sci Rep. 2018 Jul 13;8:10626. doi: 10.1038/s41598-018-27863-7 (PMC6045609; doi:10.1038/s41598-018-27863-7)
Supplement: Supplementary file 1 — Supplement [file 41598_2018_27863_MOESM1_ESM.pdf]

Supplement

Aberrant imprinting may underlie evolution of parthenogenesis

Olga Kirioukhova[1,2], Jubin N. Shah[1‡], Danaé S. Larsen[1‡], Muhammad Tayyab[1], Nora E. Mueller[1], Geetha Govind[1¶], Célia Baroux[3], Michael Federer[3], Jacqueline Gheyselinck[3], Philippa J. Barrell[3§], Hong Ma[4,5γ], Stefanie Sprunck[6], Bruno Huettel[7], Helen Wallace[8], Ueli Grossniklaus[3*] and Amal J. Johnston[1,2,3,9*]

[1]University of Heidelberg, Centre for Organismal Studies, Germline Genetics & Evo-Devo Lab, Heidelberg, Germany

[2]Jacobs University, Life Sciences & Chemistry, Germline Genetics & Evo-Devo Lab, Bremen, Germany

[3]University of Zurich, Department of Plant and Microbial Biology and Zurich-Basel Plant Science Center, Zurich, Switzerland

[4]The Pennsylvania State University, The Huck Institute of Life Sciences, Department of Biology, the University Park, Pennsylvania, USA

[5]Fudan University, State Key Laboratory of Genetic Engineering, Institute of Plant Biology, School of Life Sciences, Fudan University, Shanghai, China

[6]University of Regensburg, Cell Biology and Plant Biochemistry, Regensburg, Germany

[7]Max-Planck-Institute for Plant Breeding Research, Cologne, Germany

[8]University of the Sunshine Coast, Faculty of Science, Health, Education and Engineering, Genecology Research Centre, Maroochydore, Australia

[9]ETH Zurich, Department of Biology and Zurich-Basel Plant Science Center, Zurich, Switzerland

[¶]Present address: University of Agricultural Sciences, College of Agriculture Sciences, Department of Crop Physiology, Hassan, India

[§]Present address: New Zealand Institute for Plant and Food Research, Christchurch, New Zealand

[γ]sabbatical work at [(3)]

[‡]equally contributed

*corresponding authors. a.johnston@jacobs-university.de, amal.johnston@greentechlab.net, grossnik@botinst.uzh.ch

Supplementary Fig. 1 | Modified female meiosis ensures unreduced female germline initiation in an asexual *Boechera.*

a-k, Laser scanning confocal microscopy images of Feulgen-stained[s1] female meiotic stages in the sexual (**a-f**) and apomictic ovules (**g-k**). **a,g,** MMCs at early meiotic prophase (leptotene) with chromosomes starting to condense (arrow-heads indicate the MMC nucleus). **b,h,** MMC nucleus at meiotic metaphase I stage of the sexual species exhibits correctly paired bivalents of homologous chromosomes (**b**, arrow - 7 bright spots of bivalents), while in the apomict bivalent formation fails resulting in univalents (**h**, white arrow-head, 21 bright spots of univalents). In the sexual species, meiotic cell division I (M-I) (**c**, anaphase I, arrows) is followed by mitotic-like M-II division (**d**, metaphase II, arrows) resulting in tetrad of haploid megaspores (**e**, arrow-heads). **f,** one megaspore stays functional and gives rise to the female gametophyte (arrow-head) while three other megaspores degenerate. **i,j,k,** At the same time, the apomictic ovaries fail to form bivalents, leading to impairment of the first meiotic reduction division. **i,** The stand-alone mitotic-like division (metaphase, arrow-head). **j,** unreduced dyad formation. **k,** a functional unreduced megaspore development (arrow-head) upon degeneration of the second megaspore. Scale bars 10 µm.

<u>Additional notes to **Supplementary Fig. 1**</u>. MMC specification occurs correctly in asexual *Boechera*, as evidenced by morphological analysis. It must be noted, however, that apomeiotic ovaries might express some aberrant taxon-specific transcripts at the onset of MMC specification in preparation for apomeiosis as shown in large-scale transcriptomic studies[s2,s3]. Consistent with the diplosporous nature of apomixis proposed for most *Boecheras*[s4-s6], we noted that apomictic ovaries had meiosis I-like chromosome condensation. However, unpaired univalents were seen as products of defective synapsis of homologous chromosomes leading to direct segregation of sister chromatids. Whereas capturing female meiosis in full resolution is complicated due to difficulties in accessing ovule tissues, additional confirmation on progression of apomeiosis can be inferred by precise step-by-step analysis of male apomeiotic events pinpointing the obvious absence of pairing of homologous chromosomes during pachytene, leading to corresponding unreduced male germline formation (compare Supplementary Fig. **2f** versus **2b**, see Supplementary Fig. **2** and notes therein for details). Briefly, soma-to-germline transition in apomictic *Boechera* ovules is featured by circumvention of meiosis, likely very similar to the apomeiotic events in the corresponding male germline, resulting in diplosporous dyad formation instead of a meiotically-reduced tetrad of spores, ultimately giving rise to an unreduced *Taraxacum*-type embryo sac[s6,s7].

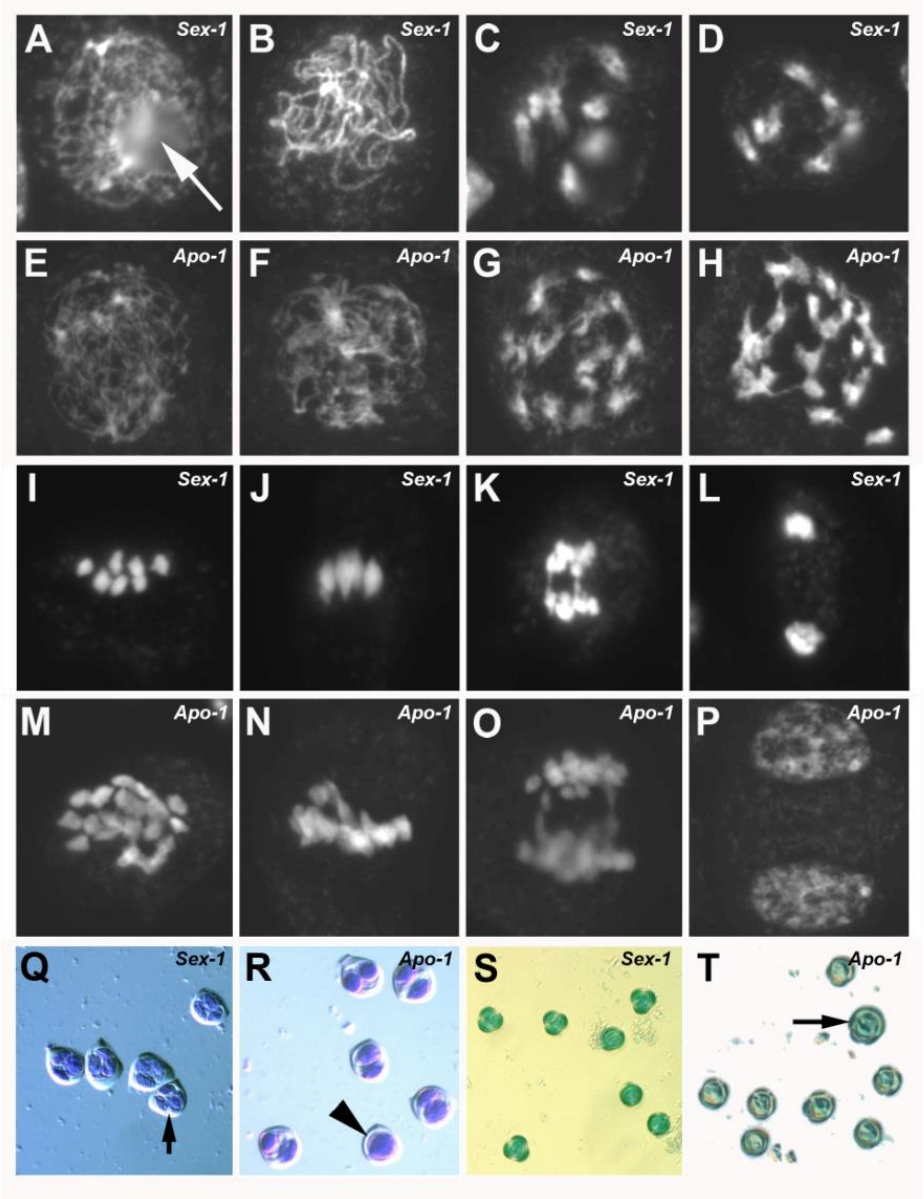

Supplementary Fig. 2 | Unreduced male germline formation in an apomictic *Boechera.*

a,b,c,d,i,j,k,l,q,s, micrographs of *Sex-1*. **e,f,g,h,m,n,o,p,r,t,** micrographs of *Apo-1*. **a,** Early prophase I, leptotene. The nucleolus is visible (arrow). **b,** Middle prophase I, pachytene, with homologs aligned and juxtaposed, forming thick thread-like chromosomes. **c,** Early diakinesis, with 7 bivalents. **d,** Late diakinesis, 7 bivalents. **e,** Early prophase I, leptotene-like, seemingly normal, but the nucleolus is not detectable. **f,** Middle prophase I. Abnormal, unlike pachytene chromosomes. **g,** Early diakinesis. **h**, Many univalents, possibly with some bivalents followed by late diakinesis. **i,** Prometaphase I, with highly condensed 7 bivalents (bright spots). **j,** Metaphase I, with bivalents aligned at the equator. **k,** Anaphase I, with separated homologs. **l,** Early telophase I, with two clusters of chromosomes that are yet to decondense. **m,** Prometaphase I. Condensed chromosomes, most of which are univalents. **n,** Metaphase I, with chromosomes aligned at the equator. **o,** Anaphase I, with separated chromosomes. **p,** Telophase I, with two clusters of decondensed chromosomes. **q,** sexual tetrahedral tetrads, with three spores visible in most cases, except one (arrow) in which all four can be seen. **r,** unreduced dyads, with two spores. One of them has only a single large spore (arrowhead). **s,** Microspores, with nearly uniform sizes. **t,** unreduced (triploid) microspores in *Apo-1*. Most have similar sizes, with occasional large one (arrow). **a-p,** are DAPI stained images of meiosis, and **q-t,** are toluidine-blue-stained DIC images.

Additional notes to **Supplementary Fig. 2**. Meiosis in *Sex-1* looked normal, with the expected stages of meiosis I (**a-d,i-l**). In particular, at mid-prophase I, typical pachytene nuclei could be found that had characteristic thick thread-like chromosomes (**b**), suggesting that pairing and synapsis between homologous chromosomes were normal. Subsequently, seven bivalents were observed at diakinesis, prometaphase I, and metaphase I (**c,d,i**), as expected for a diploid of 2n = 14 in *Sex-1 Boechera*. The homologous chromosomes then separated at anaphase I and formed two clusters at telophase I (**k,l**). In the apomictic line *Apo-1* (triploid, 3n = 21), male meiosis began seemingly normal, with leptotene-like nuclei (**e**). They had axial-like elements from condensing chromosomes which appeared as thin threads. However, classical pachytene configurations were not observed among >100 prophase I cells. Instead, abnormal meiocytes were found with chromosomes that did not seem to pair or juxtapose (**f**). As the chromosomes continue to condense, at late prophase I stages equivalent to diakinesis, it is clear that univalents were observed. At the same time, some chromosome entities were larger or brighter than others and the total number of foci was less than 21 (**g,h,m**), suggesting that there might be some bivalents. At metaphase I, the chromosomes were aligned properly (**n**). The daughter chromatids then separated at anaphase (anaphase II like) to two poles at telophase (**o**) unlike homologous chromosome segregation in the sexual anaphase I (**k**). No further nuclear division in apomicts was observed, indicating that there was only one division, and the products of the modified first division were packaged into microspores. After the completion of meiosis, the diploid *Sex-1* produced a tetrad of spores (**q**), whereas the apomict had dyads (**r**, N=600). Occasionally, meiosis in *Apo-1* produced a single large spore (**r**, arrowhead). Following the release of microspores from the dyad, the apomictic microspores (**t**) are slightly larger than the sexual microspores (**s**), because only two triploid spores are produced from each meiotic event. Also, a few spores are usually large (**t**, arrow), presumably from those apomeiotic events that yielded a single spore.

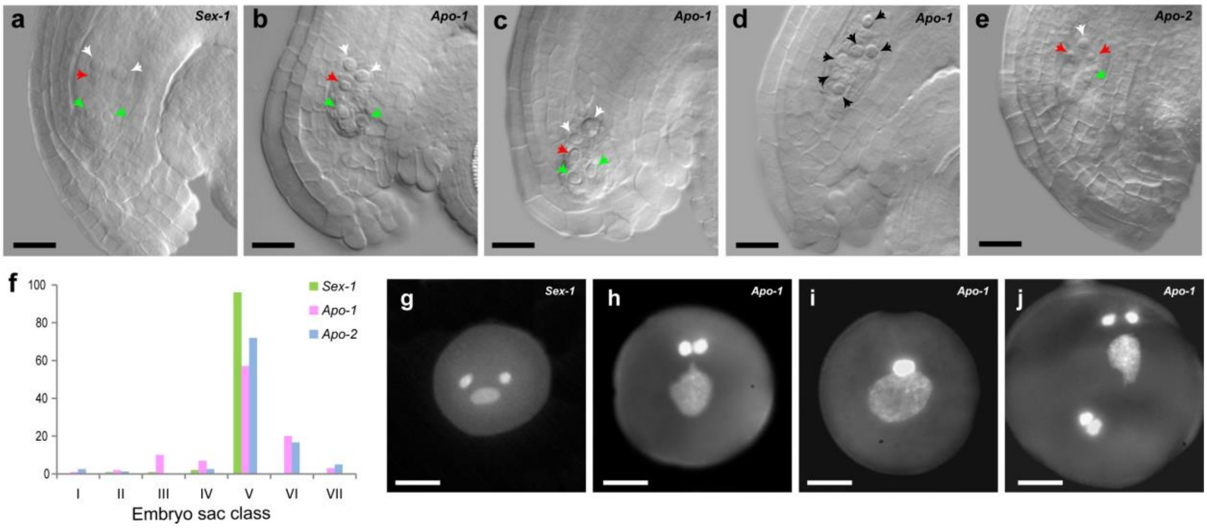

Supplementary Fig. 3 | Female and male gametophytic development in an apomictic *Boechera* compared to a corresponding sexual *Boechera*.

a-e, Terminal stages of egg cell development in sexual versus parthenogenetic ovaries. **a,** A sexual ovule showing an egg cell (red arrow head), two polar nuclei (white arrow heads) and two synergid cells (green arrow heads). **b,** Most apomictic ovaries reached the stage shown in **a**. Rare deviation included: **c,** Two polar nuclei of different size, **d,** Nuclei proliferation (black arrow heads), and **e,** Two egg-like cells. **f,** Quantification of embryo sac (ES) classes prior to fertilization events. Ovule counts: *Sex-1/Apo-1*= 104/165. ES phenotypic classes, I – VI: one–nucleate, two-nucleate, four-nucleate, eight-nucleate, mature, two polar nuclei of different size, and developmental deviations (i.e. cell proliferation, twin egg cells, collapsed etc.). **g-j**, DAPI-stained mature pollen. The majority of *Apo*-1 pollen (**h**) reached tri-cellular stage and formed two sperm cells similar to the *Sex-1* (**g**). **i,j**, Rare cases of single generative/sperm cell (**i**) or multiple sperm cells (**j**) in *Apo-1*. Scale bars in **a-e**: 20µm, in **i-l**: 10µm.

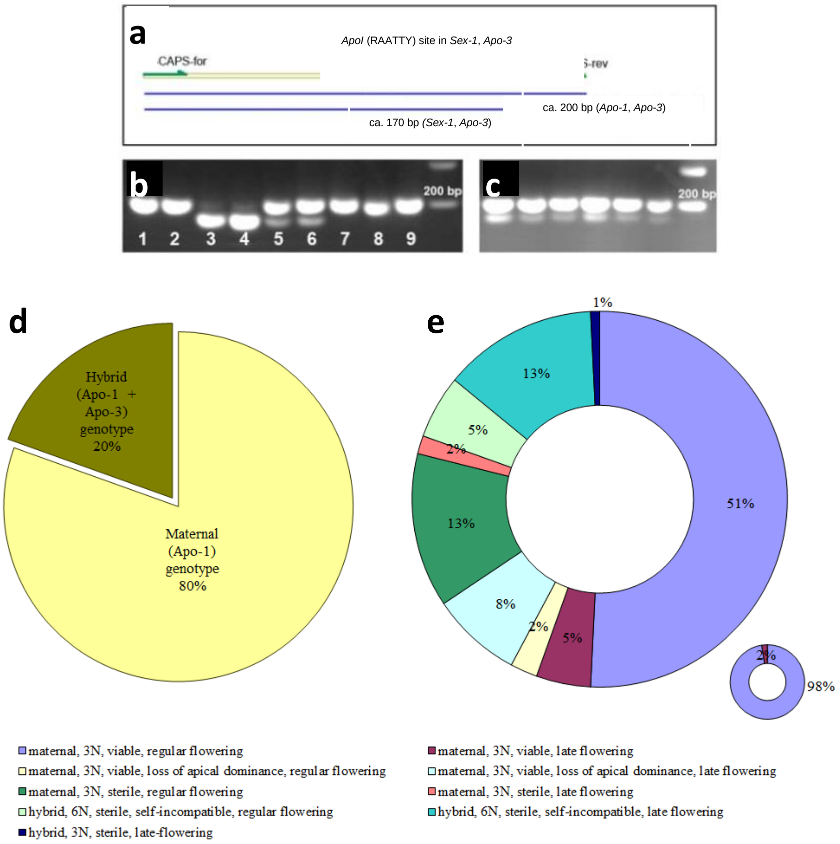

Supplementary Fig. 4 | Inter-specific hybridization of unreduced parthenogenetic gynoecia of *B. gunnisoniana* (*Apo-1*) by unreduced pollen of *B. holboellii* (*Apo-3*) leads to defective progeny development.

a, Scheme describing a CAPS marker developed to distinguish three *Boechera* genotypes: *Apo-1* (3N); *Apo-3*, (3N); and *Sex-1* (2N). **b,** Representative progeny testing of *Apo-1* × *Apo-3* using the CAPS marker. *Apo-1* mother (lanes 1,2), *Sex-1* control (lanes 3,4), *Apo-3* father (lanes 5,6) and progeny between *Apo-1* and *Apo-3* (lanes 7,8,9). **c,** Genotyping of immature F_1 seeds resulting from a cross between *Apo-1* (mother) and *Apo-3* (father) show that all the progeny seeds are of hybrid origin (N=100); "father"-specific band might be due to embryo and/or pseudogamous endosperm fertilization events. **d,** Genotypic classes of seedling progeny derived via an inter-specific cross between *Apo-1* mother and *Apo-3* father. Note that 80% of the progeny were of maternal origin, and 20% were of true hybrid origins (confirmed by CAPS and ploidy genotyping) revealing back-cross hybridization (BC; N=128). **e,** Phenotypic progeny classes of seedlings derived as above in **d**. Legends are given at the bottom of the figure. Inset chart in **e** displays the progeny classes of a selfed *Apo-1* plant (N=135).

<u>Additional notes to **Supplementary Fig. 4**.</u> The progeny test described here illustrates inter-specific hybridization barriers between apomicts. When the triploid *Apo-1* gynoecia were fertilized with pollen from triploid *Apo-3* (*B. hoelboellii*)[s6], significant numbers of off-type parthenogenetic or hybrid offspring were seen, unlike the true-to-type maternal progeny scored in self-pollinated apomictic progeny. Thus, faithful parthenogenesis in *Boechera* requires self-sperm cells.

```
Sex1_PHEL1      MR----YSLIENKTSRRTTFNKRKQGLKKKLTELVTLCDVKACGVIYSPYNPEPEAWPSREGVEEVVSEFMEVSMK
Apo1_PHEL1      MR----YSLIENKTSRRTTFNKRKQGLKKKLTELVTLCDVKACGVIYSPCNPEPEAWPSREGVEDVVSEFMEVSMK
Apo2_PHEL1      MR----YSLIENKTSRRTTFNKRKQGLKKKLTELVTLCDVKACGVIYSPYNPEPEAWPSREGVEEVVSEFMEVSMK
At_PHE1         MRGKMKLSFIENDSVRKTTFTKRKKGMLKKFNELVTLCGVDACAVIRSPYNSIQEPWPSREGVEEVMSKFMEFSVL
At_PHE2         MKRKMKLSLIENSVSRKTTFTKRKKGMTKKLTELVTLCGVEACAVVYSPFNSIPEAWPSREGVEDVVSKFMELSVL

Sex1_PHEL1      DRSKKMVDQETFLREMIAKEKAQLQKLRNENRKFQMQDIMWGCLKGEIDVRKLGEKDLRDLSSFIDKYLNQLSCRI
Apo1_PHEL1      DRSKKMVDQETFLREMIAKEKAQLQKLRNENRKFQMRYIMWGCLKGEIDVRKLGEKDLRDLSSFIDKYLNQLSCRI
Apo2_PHEL1      DRSKKMVDQETFLREMIAKEKAQLQKLRNENRKFQMRDIMWGCLKGEIDVRKLGEKDLRDLSSFIDKYLNQLSCRI
At_PHE1         DRTKKMVDQETFLRQRIAKETERLQKLRDENRNSQIRDLMFGCLKGEVDVSHLHGRDLLDLNVFLNKYLNGVIRRV
At_PHE2         DRTKKMVDQETFISQRIAKEKEQLQKLRDENHNSQIRELMFGCLKGETNVYNLDGRDLQDLSLYIDKYLNGLTRRI

Sex1_PHEL1      ENLTENGESSSSLPPLV-V-P---DALA-----------KYQKQRKLFQLQNDALLEFFGQIPKKIYDFNMNMN--
Apo1_PHEL1      KNLTENGESSSSLPPLV-V-P---DALA-----------KYQKQRKLFQLQNDALLEFFGQIPQKIHDFNMNMN--
Apo2_PHEL1      ENLTENGESSSSLPPLV-V-P---DALA-----------KYQKQRKLFQLQNDALLEFFGQIPKKIYDFNMNMN--
At_PHE1         EILKENGESSSSVPPPIGVAPTVVDASVPIGFDGRMIQDQNQNQQEPVQFQYQALYDFYDQIPKKLHDFNMKMNID
At_PHE2         EILIENGESSSSLPLPI-V-A---NAAAPVGFDGPMFQYHNQNQQKPVQFQYQALYDFYDQIPKKIHGFNMNMNKD

Sex1_PHEL1      SNQNMILDLNQDLNVEEDGDIPSMDGSHHQPETDCLAA-TAA--DACAPNITNNLES*
Apo1_PHEL1      SNQNMILDLNQDLNVEEDGDILSMDGSHHQPETDCLAA-TAA--DACAPNITNNLES*
Apo2_PHEL1      SNQNMILDLNQDLNVEEDGDNPSMDGSHHQPETDCLAA-TAA--DACAPNITNNLES*
At_PHE1         PNQSMNLD----LNDGEDEGIPCMDNNNYHPEIDCLATVTTAPTDVCAPNIINDL*
At_PHE2         SNQSMVLDLNQNLNDGEDEGIPCMDNNNYHPEIDCLATVTTAPTDVCAPNITNDL*
```

Supplementary Fig. 5 | The *Boechera* PHEL1 sequence compared to the *Arabidopsis* PHE1 and PHE2

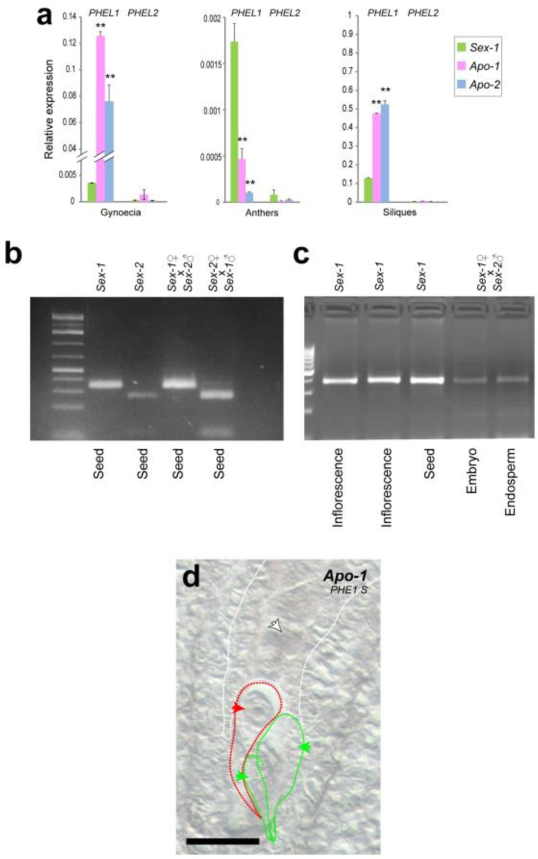

Supplementary Fig. 6 | Parental *PHEL1* expression.

(a) In contrast to *PHEL1*, *PHEL2* expression is not faithfully detectable by qRT-PCR. **(b,c)** Maternal and paternal *PHEL1* transcripts assayed by allele-specific RT-PCR in immature seeds **(a)** and in embryo and endosperm fractions **(b)** upon reciprocal crosses between two sexual *Boechera* accessions. Full-length gel images. **(d)** Negative mRNA *in situ* control with a sense *PHE1* probe did not show a signal in *Apo-1* ovule. Color-codes as in Fig. 1.

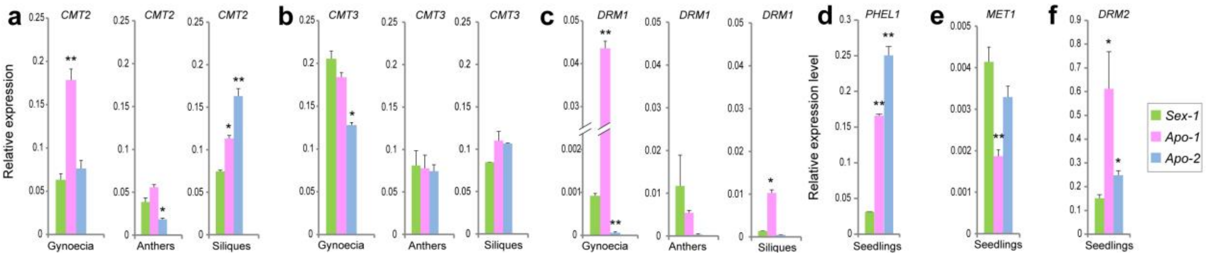

Supplementary Fig. 7 | Relative gene expression.

Relative transcript levels of *CMT2* (**a**), *CMT3* (**b**) and *DRM1* (**c**) in reproductive tissues, and *PHEL1* **(d),** *MET1* **(e)** and *DRM2* **(f)** in vegetative tissues (seedlings) of *Boechera*. t-test significance levels for difference of expression between an apomict and sexual: **$\alpha \leq 0.01$;*$\alpha \leq 0.05$.

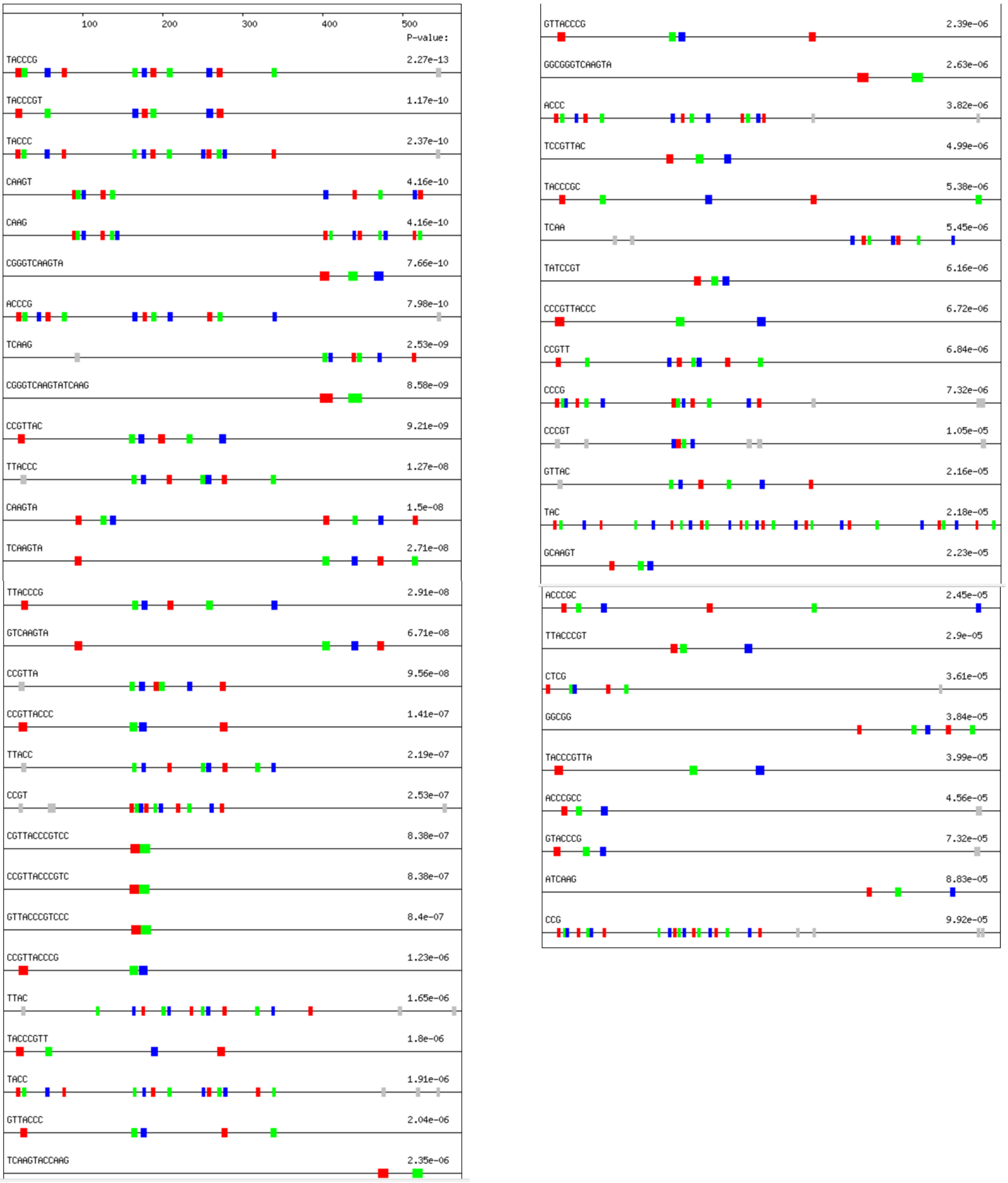

Supplementary Fig. 8 | The 3’ methylated region (3’ MR) of the *Boechera PHEL1* locus contains several repeat motifs exclusively in the sexually reproducing plants.

Shown is a panel of several repeats identified by REPFIND (https://zlab.bu.edu/mfrith/cgi-bin/repfind.cgi) in the sequence unique for the sexual species *Sex-1*.

Supplementary Table 1 |. List of primers used in this study.

Gene	Forward primer	Reverse primer	Purpose	Ref
RPS18	*GCTGGGGAGTTATCTGCTGCTGAG*	*CTTGCCGTCTTTGTAATCCTTCTGC*	qRT-PCR	s8
PHEL1	*GTTGAAGAGGATGGAGACATTC*	*TGCAGACGATTAAATACTTCACTC*	qRT-PCR	
PHEL2	*CGGTTAGAGAGCAGACCAAG*	*CTCGAAGATCCCTTTGATCAAGA*	qRT-PCR	
MEA	*CTGCTCATGCTTCACATCATCAA*	*CATAATTAGAGGAGCCACTGAAATG*	qRT-PCR	
MET1	*GGTGCCGATACTCGTTGATAA*	*CATTGCTTGCTTGATTGATGAAC*	qRT-PCR	
DRM1	*AGTCGCACTTCATCGTCTCA*	*ACGGACTTCCTCCTATGACC*	qRT-PCR	
DRM2	*TCAGACGTCCAAAACTTGACCA*	*CTCGGCTTACCCTGTTACCG*	qRT-PCR	
CMT2	*CAACCGTTCTAACAGTCCCAA*	*GAGACTGCCACTGCATTTCC*	qRT-PCR	
CMT3	*GACTCCAGGGCTTTCCTGATT*	*TCCCAAAGCATATCCAAGGGC*	qRT-PCR	

Supplementary references

s1 Barrell, P. & Grossniklaus, U. Examining female meiocytes of maize by confocal microscopy. *Methods Mol. Biol.* **990**, 45-52, doi:10.1007/978-1-62703-333-6_5 (2013).

s2 Schmidt, A. et al. Apomictic and sexual germline development differ with respect to cell cycle, transcriptional, hormonal and epigenetic regulation. *PLoS Genet.* **10**, e1004476, doi:10.1371/journal.pgen.1004476 (2014).

s3 Sharbel, T. F. et al. Apomictic and sexual ovules of *Boechera* display heterochronic global gene expression patterns. *Plant Cell* **22**, 655-671, doi:10.1105/tpc.109.072223 (2010).

s4 Böcher, T. W. Cytological studies of *Arabis holboellii*. *Hereditas* **32**, 573-575 (1947).

s5 Roy, B. A. The breeding systems of six species of *Arabis* (Brassicaceae). *Am. J. Bot.* **82**, 869-877 (1995).

s6 Naumova, T. N. et al. Reproductive development in apomictic populations of *Arabis holboellii* (Brassicaceae). *Sex. Plant Reprod.* **14**, 195-200, doi:http://www.springerlink.com/content/l2dxbmaducx3y5wy/ (2001).

s7 Crane, C. F. in The flowering of apomixis: From mechanisms to genetic engineering (eds Y. Savidan, J. G. Carman, & T. Dresselhaus) Ch. 24-43, 168-211 (CIMMYT, IRD, European Commission DG VI (FAIR), 2001).

s8 Pellino, M., Sharbel, T. F., Mau, M., Amiteye, S. & Corral, J. M. Selection of reference genes for quantitative real-time PCR expression studies of microdissected reproductive tissues in apomictic and sexual *Boechera*. *BMC research notes* 4, 303, doi:10.1186/1756-0500-4-303 (2011).
